# Supplementary material for: Impact of Long-Term Statin Therapy on Incidence and Severity of Community-Acquired Pneumonia: A Real-World Data Analysis
Source: Biomedicines. 2025 Jun 11;13(6):1438. doi: 10.3390/biomedicines13061438 (PMC12189959; doi:10.3390/biomedicines13061438)
Supplement: Supplementary file 1 [file biomedicines-13-01438-s001.zip › biomedicines-3638815-supplementary.pdf]

Impact of Long-Term Statin Therapy on Incidence and Severity of Community-Acquired Pneumonia: A Real-World Data Analysis

Supplementary Materials

Diana Toledo[1,2], Àurea Cartanyà-Hueso[3], Rosa Morros[3,4,5], Maria Giner-Soriano[3,6], Àngela Domínguez[1,2], Carles Vilaplana-Carnerero[1,7], María Grau[1,2]*

1 Department of Medicine, School of Medicine and Health Sciences, University of Barcelona, 08036 Barcelona, Spain
2 Biomedical Research Consortium in Epidemiology and Public Health (CIBERESP), 28029 Madrid, Spain
3 Fundació Institut Universitari per a la Recerca a l'Atenció Primària de Salut Jordi Gol i Gurina (IDIAPJGol), 08007 Barcelona, Spain
4 Biomedical Research Consortium in Infectious Diseases (CIBERINFEC), 28029 Madrid, Spain
5 Department of Pharmacology, Therapeutics and Toxicology, School of Medicine, Universitat Autònoma de Barcelona, 08193 Bellaterra, Spain
6 School of Medicine, Universitat Autònoma de Barcelona, 08193 Bellaterra, Spain
7 Service for the Promotion of Quality and Bioethics, General Directorate of Health Planning and Regulation, Department of Health, Government of Catalonia, 08028 Barcelona, Spain
* Correspondence: mariagrau@ub.edu

Supplementary Figure S1. Standardized Mean Differences (SMDs) for all covariates before and after matching.

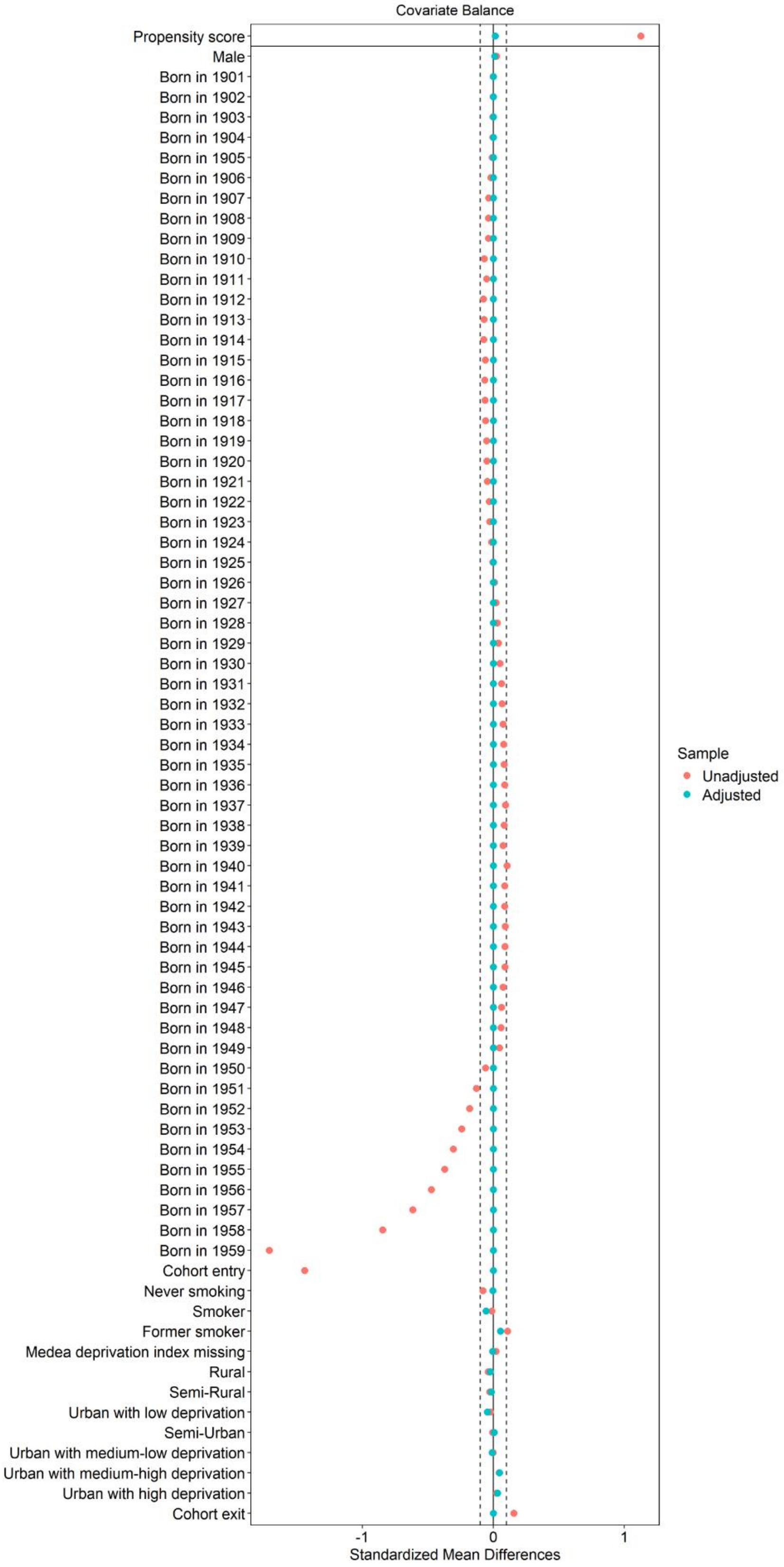

Supplementary Table S1. Summary of Models 1–6 with Increasing Covariate Adjustment

	**Model 1**	**Model 2**	**Model 3**	**Model 4**	**Model 5**	**Model 6**
	Crude risk ratio (95% CI)	Adjusted risk ratio (95% CI)	Adjusted risk ratio (95% CI)	Adjusted risk ratio (95% CI)	Adjusted risk ratio (95% CI)	Adjusted risk ratio (95% CI)
CAP incidence	1.15 (1.12;1.18)	0.93 (0.91;0.96)	0.94 (0.91;0.96)	0.98 (0.95;1.01)	0.98 (0.95;1.00)	0.97 (0.95;1.00)
CAP severity (ICU admission)	1.15 (1.09;1.20)	0.92 (0.87;0.97)	0.93 (0.88;0.98)	0.98 (0.93;1.03)	0.98 (0.93;1.03)	0.97 (0.92;1.02)

CAP: Community acquired pneumonia. CI: Confidence interval. ICU: Intensive care unit

Model 2 adjusted for Charlson index

Model 3 adjusted for Charlson index and pneumococcal vaccine

Model 4 adjusted for Charlson index, diabetes, hypercolesterolemia, obesity and hypertension

Model 5 adjusted for Charlson index, pneumococcal vaccine, diabetes, hypercolesterolemia, obesity and hypertension

Model 6 adjusted for Charlson index, pneumococcal vaccine, diabetes, hypercolesterolemia, obesity, hypertension and smoker

Supplementary Table S2. Risk ratios for all variables included in the adjusted models

**Outcome**		**Model 1**	**Model 2**	**Model 3**	**Model 4**	**Model 5**	**Model 6**
CAP incidence		Crude risk ratio (95% CI)	Adjusted risk ratio (95% CI)	Adjusted risk ratio (95% CI)	Adjusted risk ratio (95% CI)	Adjusted risk ratio (95% CI)	Adjusted risk ratio (95% CI)
	Statins use (ref: No)	1.15 (1.12,1.18)	0.93 (0.91,0.96)	0.94 (0.91,0.96)	0.98 (0.95,1.01)	0.98 (0.95,1.00)	0.97 (0.95,1.00)
	Charlson Index		1.25 (1.24,1.26)	1.25 (1.24,1.26)	1.24 (1.23,1.25)	1.24 (1.23,1.26)	1.23 (1.22,1.24)
	Pneumococcal vaccination (ref: No)			0.54 (0.5,0.57)		0.6 (0.56,0.64)	0.59 (0.55,0.63)
	Diabetes (ref: No)				0.98 (0.94,1.02)	1 (0.96,1.04)	0.98 (0.94,1.02)
	Hypertension (ref: No)				1.01 (0.98,1.04)	1.03 (1,1.06)	1.03 (1,1.06)
	Hypercholesterolemia (ref: No)				0.67 (0.64,0.69)	0.69 (0.66,0.71)	0.69 (0.67,0.72)
	Obseity (ref: No)				0.88 (0.85,0.92)	0.91 (0.87,0.95)	0.91 (0.87,0.95)
	Former smoker (ref: Non-smoker)						1.58 (1.53,1.63)
	Smoker (ref: Non-smoker)						1.32 (1.27,1.38)
ICU Admission							
	Statins use (ref: No)	1.14 (1.09,1.2)	0.92 (0.87,0.97)	0.93 (0.88,0.98)	0.98 (0.93,1.03)	0.98 (0.93,1.03)	0.97 (0.92,1.02)
	Charlson Index		1.28 (1.27,1.3)	1.29 (1.27,1.31)	1.28 (1.26,1.3)	1.28 (1.26,1.3)	1.27 (1.25,1.29)
	Pneumococcal vaccination (ref: No)			0.40 (0.34,0.46)		0.46 (0.4,0.54)	0.45 (0.39,0.53)
	Diabetes (ref: No)				0.94 (0.87,1.02)	0.97 (0.89,1.05)	0.95 (0.87,1.03)
	Hypertension (ref: No)				0.96 (0.91,1.02)	0.99 (0.93,1.05)	1.00 (0.94,1.06)
	Hypercholesterolemia (ref: No)				0.59 (0.55,0.64)	0.62 (0.57,0.66)	0.62 (0.58,0.67)
	Obseity (ref: No)				0.88 (0.81,0.96)	0.92 (0.84,1.00)	0.91 (0.84,0.99)
	Former smoker (ref: Non-smoker)						1.72 (1.61,1.83)
	Smoker (ref: Non-smoker)						1.38 (1.28,1.49)

ICU: Intensive Care Unit. Ref: reference
